# Pattern of Skin Diseases at a Dermatology Center: A Retrospective Study

**DOI:** 10.7759/cureus.65259

**Published:** 2024-07-24

**Authors:** Suhasini Krishnan, Khalifa Almheiri

**Affiliations:** 1 Medicine, Dubai Academic Health Corporation, Dubai, ARE; 2 Dermatology, Dubai Health, Dubai , ARE

**Keywords:** outpatient clinic, outpatient, dermatology, dermatological disorders, prevalence

## Abstract

Introduction: Factors such as location, climate, dietary habits, and socioeconomic status as well as age and gender all directly influence the development of certain skin disorders. Whilst the pattern of skin diseases has been studied in the Middle East region previously, data on the incidence of these conditions in the United Arab Emirates (UAE) is lacking. This retrospective study aims to identify the types and frequency of dermatological disorders encountered at a dermatology outpatient clinic in Dubai, UAE.

Methods: Electronic medical records obtained from the outpatient clinic of the Dermatology Department at Rashid Hospital, from January 1, 2021, to January 1, 2022, were retrospectively assessed. We only included new patients who visited the clinic for the first time. Patient data was analyzed based on their clinical diagnosis and were then grouped as per age, gender, and diagnosis.

Results: A total of 5969 new patient encounters were recorded in 12 months, a majority of which were female patients, 3526 visits (n=3526, 59.1%). The frequency of visits made by male patients was 2443 (n=2443, 40.9%). The 10 most frequently encountered conditions were the following: acne (17.6%), unspecified dermatitis (9.9%), atopic dermatitis (5.5%), viral warts (4.5%), seborrheic dermatitis (4.3%), psoriasis (4.2%), dermatophytosis (3.5%), xerosis cutis (3.1%), non-scarring hair loss (2.1%), rash and other non-specific skin eruption (2.1%).

Conclusion: Acne was the most prevalent skin condition seen in the clinic year-round, followed by dermatitis. The pattern of skin diseases can be a good indicator for community health and in planning preventative and therapeutic strategies. Involving primary care physicians in the management of these conditions can lead to an earlier diagnosis and management, thereby improving the quality of patients’ lives.

## Introduction

Globally, identifying the pattern of skin diseases has become of increasing interest. These epidemiological studies help identify those conditions that are easily transmissible, treatable, and most importantly preventable [1]. Although these conditions are rarely life-threatening, except for a few, they can still have significant adverse effects on the patient’s physical and mental well-being, as well as pose a significant economic burden on primary health care and dermatology centers. Prevention strategies such as improving sanitation and patient education regarding hygiene and nutrition can help reduce the burden in community-based healthcare centers [2,3].

In developed countries, it has been estimated that one in three patients presents with a dermatological complaint at any point in time. To note, in developing countries, infectious diseases were more prevalent [1]. Dermatological conditions are one of the main presenting complaints seen in primary healthcare centers, accounting for 14% of visits [2]. The pattern, prevalence, and incidence of these skin conditions depend not only on the patient’s ethnicity and genetic make-up but also on other factors such as hygiene, dietary habits, traditions and customs, and lastly, the climate of the particular geographical location [1,2]. This study was performed in the city of Dubai, which is located in the United Arab Emirates (UAE), with a population of 3.49 million. The average air temperature value in the last 10 years measured between + 31°C and + 40°C [4]. Dubai has primarily two main seasons, winter and summer, with a few transient periods of varying weather in between. The winter months are from December to March, and the summer season is mainly from June to September [5]. Whilst studies assessing the pattern of skin diseases have been conducted in the Middle East region, primarily Saudi Arabia, literature on the UAE is scarce [2,6-9]. This study aims to identify the types and frequency of dermatological disorders that appear at a dermatology outpatient clinic, taking into account the age and gender of the patients, as well as the climate. 

## Materials and methods

This is a retrospective study, conducted over a year, from January 1, 2021, to January 1, 2022, and was approved by the Dubai Scientific Research Ethics Committee (Reference no. DSREC-12/2022_13). Inclusion criteria consisted of new patients first presenting to the Dermatology Department’s outpatient clinic in Rashid Hospital, Dubai, UAE. Patients’ data, collected from electronic medical records, were summarized and analyzed based on their clinical diagnosis, as well as histopathological and laboratory investigations. The diagnosis was categorized into 15 categories according to their class and further classified into the following to make the study more comprehensive as per the International Statistical Classification of Diseases and Related Health version 10 (ICD 10) [10]: diseases of the skin and subcutaneous tissue, infectious and parasitic diseases, neoplasms, congenital malformations, deformations, chromosomal abnormalities, and diseases of the oral cavity, salivary glands and jaws. Complaints that did not fit into any of the above were classified into the group of others. We also reviewed similar articles conducted in Saudi Arabia to assess how the diseases were categorized [2,6,7]. Patients were grouped into three categories according to their age; those between the ages of 0 to 18 years were children and those between 19 to 69 years were adults, with the remaining considered older adults. The data was entered into IBM SPSS Statistics for Windows, Version 23 (Released 2015; IBM Corp., Armonk, New York, United States) to calculate the frequency of the skin diseases in total and on the basis of age and sex differences. With the dates of the summer and winter months of Dubai established, data was analyzed to identify the five most prevalent conditions during these times [5].

## Results

As seen in Table 1, a total of 5969 patient encounters were recorded in 12 months at the dermatology outpatient clinic, a majority of which were female patients, 3526 visits (n=3526, 59.1%). The frequency of visits made by male patients was 2443 (n=2443, 40.9%).

**Table 1:** The frequency of males and females attending the outpatient clinic.

S No	Gender	Frequency (n)	%
1	Male	2443	40.9%
2	Female	3526	59.1%
3	Total	5969	100

The pattern of skin diseases seen at the dermatology outpatient clinic is described in Table 2. The dermatome diseases were divided into 15 categories as per ICD 10. Of these, skin disorders of the appendages (n=1849, 30.9%), dermatitis, and eczema (n=1249, 20.9%), as well as infections and parasitic diseases (n = 747, 12.5%), constituted the most common skin disorders encountered. Specifically, acne (n=1050, 17.6%) was the most common diagnosis, followed by unspecified dermatitis (n=595, 9.9%), and then atopic dermatitis (AD) (n = 328, 5.5%).

**Table 2:** The frequencies and rates of the disease groups.

Diseases			Patients (n)	%
Diseases of the skin and subcutaneous tissue	1. Skin disorders of the appendages	Acne	1050	17.60%
		Rosacea	47	0.80%
		Follicular disorder, unspecified	48	0.80%
		Perifolliculitis capitis abscedens	6	0.10%
		Folliculitis decalvans	2	0.03%
		Alopecia	2	0.03%
		Non-scarring hair loss	127	2.10%
		Telogen effluvium	19	0.30%
		Androgenic alopecia	24	0.40%
		Alopecia areata	75	1.30%
		Hirsutism	7	0.10%
		Trichotillomania	2	0.03%
		Nail disorder, unspecified	12	0.20%
		Hidradenitis suppurativa	46	0.80%
		Seborrheic keratosis	31	0.50%
		Xerosis cutis	183	3.10%
		Rash and other non-specific skin eruption	122	2.00%
		Hypertrophic scar	18	0.30%
		Primary focal hyperhidrosis, palms	18	0.30%
		Stevens-Johnson syndrome	5	0.10%
		Vesicular eruption, localized	1	0.02%
		Striae atrophicae	4	0.10%
	2. Dermatitis and Eczema	Dermatitis, unspecified	595	9.90%
		Atopic dermatitis	329	5.50%
		Seborrheic dermatitis	258	4.30%
		Lichen simplex chronicus	15	0.30%
		Pruritus	47	0.80%
		Nodular prurigo	5	0.10%
	3. Papulosquamous disorders	Psoriasis	256	4.30%
		Lichen planus	23	0.40%
		Pityriasis rosea	18	0.30%
		Other papulosquamous disorders	1	0.02%
	4. Pigmentary disorders	Vitiligo	102	1.70%
		Acanthosis nigricans	13	0.20%
		Other melanin hyperpigmentation	82	1.40%
		Other disorders of pigmentation	18	0.30%
		Post-inflammatory hyperpigmentation	13	0.20%
		Albinism	1	0.02%
		Pityriasis alba	15	0.30%
		Chloasma	87	1.50%
		Freckles	4	0.10%
		Leukoderma	1	0.02%
	5. Urticaria and erythema	Urticaria	113	1.90%
		Erythema intertrigo	36	0.60%
		Erythema multiforme	6	0.10%
		Erythema annulare centrifugum	2	0.03%
		Other erythematous conditions	6	0.10%
	6. Bullous disorders	Pemphigus	12	0.20%
		Dermatitis herpetiformis	26	0.40%
		Bullous pemphigoid	5	0.08%
	7. Radiation-related disorders of the skin and subcutaneous tissue	Polymorphous light eruption	2	0.03%
		Sunburn	2	0.03%
		Other specified acute skin changes due to ultraviolet radiation	19	0.30%
Infectious and parasitic diseases	8. Viral infections	Zoster	15	0.30%
		Viral warts	269	4.50%
		Herpes Simplex	13	0.20%
		Molluscum contagiosum	40	0.70%
	9. Mycoses	Dermatophytosis	209	3.50%
		Pityriasis versicolor	71	1.20%
		Candidiasis	10	0.20%
		Mycosis	25	0.40%
	10. Bacterial infections	Cutaneous abscesses, furuncles and carbuncles	36	0.60%
		Impetigo	8	0.10%
		Syphilis	3	0.05%
		Cellulitis	14	0.20%
		Erythrasma	7	0.10%
		Pyoderma	4	0.06%
	11. Parasitic infections	Scabies	17	0.30%
		Pediculosis	5	0.10%
		Taeniasis	1	0.02%
Neoplasms	12. Benign	Melanocytic naevi	35	0.60%
		Hemangioma	12	0.20%
		Pyogenic granuloma	1	0.02%
		Neoplasm of unspecified behavior of unspecified site	3	0.05%
	13. Malignant	Basal cell carcinoma	6	0.10%
		Mycosis fungoides	9	0.20%
		Non-Hodgkin lymphoma	1	0.02%
		Melanoma	1	0.02%
14. Congenital malformations, deformations and chromosomal abnormalities		Neurofibromatosis	1	0.02%
		Harlequin fetus	4	0.06%
		Ichthyosis vulgaris	6	0.10%
		Congenital non-neoplastic nevus	6	0.10%
15. Diseases of the oral cavity, salivary glands and jaws		Dry mouth	1	0.02%

Table 3 describes the distribution of the 10 most common diseases encountered according to gender. Overall, female patients made up most of the encounters as compared to male patients. With regard to acne, the number of females (n=805, 76.7%) tripled that of the number of males (n=244, 23.2%). Just over half of all the patients diagnosed with unspecified dermatitis were females (n=330, 55.4%), with male patients constituting the remaining (n=265, 44.5%). In the case of AD, 170 male patients (n=170, 51.6%) were diagnosed, with 159 females making up the rest (n=159, 48.3%).

**Table 3:** The distribution of the 10 most common diseases according to gender.

S No.	Disease	Total		Male		Female	
		n	%	n	%	n	%
1	Acne	1050	17.60%	244	23.20%	805	76.7%
2	Unspecified dermatitis	595	9.90%	265	44.50%	330	55.4%
3	Atopic dermatitis	329	5.50%	170	51.6%	159	48.3%
4	Viral warts	269	4.50%	154	57.2%	115	42.7%
5	Seborrheic dermatitis	258	4.30%	113	43.7%	145	56.2%
6	Psoriasis	256	4.20%	131	51.1%	125	48.8%
7	Dermatophytosis	209	3.50%	113	54%	96	45.9%
8	Xerosis cutis	183	3.10%	84	45.9%	99	54.0%
9	Non-scarring hair loss	127	2.10%	37	29.1%	90	70.8%
10	Rash and other non-specific skin eruption	122	2.10%	55	45.0%	67	54.9%

Table 4 describes the distribution of the 10 most common diseases with regard to the age of the patients. Among the 1050 diagnosed cases of acne, adults between the ages of 19 and 69 years constituted more cases (n=630, 60.1%) followed by children between 0 and 18 years of age (n=420, 40.03%). Regarding unspecified dermatitis, 351 encounters belonged to adults (n=351, 58.9%), 216 children were diagnosed with the same (n=216, 36.3%), and the remaining constituted older adults, aged 70 years and above (n=28, 4.7%). Two hundred and sixty-three children were diagnosed with AD (n=263, 79.9%), with adults (n=65, 19.7%) and older adults (n=1, 0.3%) constituting the remainder.

**Table 4:** The distribution of the 10 most common diseases according to age.

S No.	Diseases	Total		Children (0-18 years)		Adults (19-69 years)		Older Adults (>= 70 years)	
		n	%	n	%	n	%	n	%
1	Acne	1050	17.60%	420	40.03%	630	60.1%	0	0%
2	Unspecified dermatitis	595	9.90%	216	36.30%	351	58.90%	28	4.7%
3	Atopic dermatitis	329	5.50%	263	79.90%	65	19.70%	1	0.3%
4	Viral warts	269	4.50%	96	35.60%	169	62.80%	4	1.5%
5	Seborrheic dermatitis	258	4.30%	111	43.02%	142	55%	5	1.9%
6	Psoriasis	256	4.20%	39	15.20%	216	84.30%	1	0.3%
7	Dermatophytosis	209	3.50%	54	25.80%	140	66.90%	15	7.1%
8	Xerosis cutis	183	3.10%	68	37.10%	91	49.70%	24	13.1%
9	Non-scarring hair loss	127	2.10%	40	31.40%	87	68.50%	0	0%
10	Rash and other non-specific skin eruption	122	2.10%	33	27.04%	84	68.80%	5	4.1%

Table 5 shows the frequency of the five most common diseases observed during the winter season, of which acne (n=298, 44.5%), unspecified dermatitis (n=154, 23.7%), and psoriasis (n=68, 10.5%) were the most commonly encountered diseases. 

**Table 5:** The frequency of the five most common diseases during the winter season.

S No	Diseases	Patients (n)	%
1	Acne	298	44.5%
2	Dermatitis, unspecified	154	23.7%
3	Psoriasis	68	10.5%
4	Other disorders of skin and subcutaneous tissues, not elsewhere classified	65	10.0%
5	Seborrheic dermatitis	64	9.9%

Table 6 gives information on the frequency of the five most common disease conditions during the summer months. Acne (n=360, 44.04%), unspecified dermatitis (n=190, 24.6%), and viral warts (n=91, 11.8%) were among the most commonly diagnosed conditions during this period.

**Table 6:** The frequency of the five most common diseases during the summer season.

S No	Diseases	n	%
1	Acne	340	44.04%
2	Dermatitis, unspecified	190	24.6%
3	Viral warts	91	11.8%
4	Seborrheic dermatitis	76	9.8%
5	Xerosis Cutis	75	9.7%

## Discussion

Upon conducting a review of the literature available on the pattern of skin diseases in various countries, it has been noted that there tends to be a female preponderance at the dermatology clinic. This point has been emphasized and discussed in similar studies conducted in Saudi Arabia, Turkey as well as other areas such as Pakistan and Uganda [2,3,6-8,11,12]. The presumption is that female patients are more conscious of their skin health and could demonstrate more knowledge and awareness of dermatological diseases [2,3,6,7].

In one study by Mahfouz et al., the prevalence of acne has been reported to be the eighth most prevalent disease, around 9.4%, on a global scale [13]. In our study, acne (n=1050, 17.6%) was the most commonly diagnosed condition at our center, with a prevalence of 17.6%. Our data found a strong female preponderance (n=805, 76.6%), nearly 76.6%. This finding could also be because more females may be affected by skin disorders due to multifactorial reasons such as hormonal imbalances, use of cosmetics, stress, and so on [2,3]. For example, in postmenopausal patients, studies have reported that due to the decreased estrogen, skin thickness may be affected, causing a thinning of the epidermal layer [14,15]. It has been theorized that there has been an increasing number of women of reproductive age diagnosed with polycystic ovarian syndrome, of which acne is an undesired consequence of the hyperandrogenic state [16]. This is, however, a possibility, as our data did not report on whether or not the patients were afflicted with comorbid conditions.

In our study, 595 patients were diagnosed with unspecified dermatitis (n=595, 9.9%) at the outpatient clinic. According to the ICD 10, this diagnosis is an umbrella term, encompassing those conditions wherein the skin becomes inflamed and blisters leading to the formation of a thick, scaly crust. Of these, AD is the most common type [17]. The global prevalence of AD has been reported to be 2.6, which is nearly 204.05 million people, 102.78 million being children [18]. Supporting this estimation, our data found that almost 80% of children presenting to the outpatient clinic were diagnosed with AD (n=263, 79.9%). Other studies conducted worldwide, especially in the Middle East, had similar findings, with AD being one of the most commonly diagnosed conditions among children [1-3,6]. An increasing trend in the prevalence of AD has been previously reported in the Gulf Peninsula region, however, literature studying this issue has been scarce. In a study by Mahmoud et al., the prevalence of AD in the UAE has been reported to be 16.7% [19]. Our study found that in total 329 patients (n=329, 5.5%) were diagnosed with AD. A study by Al Hammadi et al. found that the prevalence of AD in Dubai was reported to be between 4 and 5%, similar to our study’s findings [20].

Seasonal variations have been shown to affect skin health. Changes in temperature, humidity, moisture, and ultraviolet radiation exposure are some of the factors that can adversely impact the epidermal barrier [6]. Hancox et al. studied the seasonal variation of dermatological conditions in the USA and found that climate had a significant effect on skin health and could predispose patients to develop certain skin disorders [21]. In our study, during the winter months, we found that acne (n=298, 44.5%), dermatitis (n=154, 23.7%), and psoriasis (n=68, 10.5%) were the most commonly diagnosed conditions. Our findings were similar to those reported by Hancox et al., who mentioned that both seborrheic dermatitis and psoriasis have been shown to worsen in the winter and improve in the summer. They also found that acne cases were more in number in the winter as compared to the summer months, and theorize that young people, who are most likely to be affected by the condition and attend school more frequently in the winter months, would seek physician advice during this time. They also mention the use of heavier, tighter clothing in the colder season, which could exacerbate acne [21]. In the summer months, our data showed that whilst acne (n=340, 44.0%) and dermatitis (n=190, 24.6%) were still the most prevalent conditions, viral warts (n=91, 11.8%) were increasingly encountered during this time. In a study discussing the prevalence of common viral skin infections in beach volleyball athletes, they discuss that exposure to sunlight, wearing tight clothing, persistent sweating, and high moisture environments create a highly favorable environment for the growth of viral warts [22]. A surprising finding in our study is the increased prevalence of xerosis cutis (n=75, 9.7%) during the summer season. Although exposure to cold weather is known to exacerbate the condition, intense light and dehydration either due to decreased water intake or increased perspiration can also worsen the condition [23].

This study presents with some limitations. Firstly, we have only estimated the distribution and pattern of skin diseases for a period of one year, from 2021 to 2022. There could be variations in the pattern in either the previous or following years. Secondly, studies reporting seasonal variability of skin conditions in the Middle East region are few; therefore, there is limited data to compare our findings to. In addition to this, whilst similar studies have been conducted in the Middle East, literature specific to the UAE is scarce. To identify any patterns in the variation of skin disorders, we recommend further studies be conducted with a larger patient population, over a longer period, including analyzing any seasonality of these visits.

## Conclusions

The assessment of the pattern of skin diseases in a region can be indicative of the health of a community and therefore can be a useful tool in planning effective preventative and therapeutic strategies. Such studies in the UAE have rarely been conducted. In our study at a dermatology outpatient clinic, we found acne to be the most commonly encountered condition year-round, followed by dermatitis. We believe that it would be helpful to introduce some training courses for primary care physicians and general practitioners on the management of these frequently diagnosed dermatological conditions so that patients may receive an earlier diagnosis and in turn earlier treatment and referrals to specialist care when required. This can improve the quality of patients’ lives.
